# Evidence-based policy making for health promotion to reduce the burden of non-communicable diseases in Moldova

**DOI:** 10.1186/s12919-020-0183-8

**Published:** 2020-03-06

**Authors:** Florence Sécula, Séverine Erismann, Carolina Cerniciuc, Angel Chater, Lion Shabab, Fiona Glen, Ala Curteanu, Aliona Serbulenco, Natalia Silitrari, Daniela Demiscan, Helen Prytherch

**Affiliations:** 1 Swiss Tropical and Public Health Institute, P.O. Box, CH-4002 Basel, Switzerland; 2 University of Basel, P.O. Box, CH-4003 Basel, Switzerland; 3 Swiss Development Cooperation’s Healthy Life Project, Chisinau, Republic of Moldova; 4 Department of Psychology and Sport Sciences, University of Hertfordshire, Hatfield, AL10 9AB UK; 5 School of Sport Science and Physical Activity, Centre for Health, Wellbeing and Behaviour Change, University of Bedfordshire, Bedford, MK41 9EA UK; 6 UCL School of Pharmacy, Centre for Behavioural Medicine, Research Department of Practice and Policy, University College London, BMA House, London, WC1H 9JP UK; 7 Department of Behavioural Science and Health, University College London, 1-19 Torrington Place, London, WC1E 6BT UK; 8 National Institute for Health and Care Excellence, 10 Spring Gardens, London, SW1A 2BU UK; 9 Ministry of Health, Labor and Social Protection of the Republic of Moldova, Subsequently Nicolae Testemitanu State University of Medicine and Pharmacy of the Republic of Moldova, Chisinau, Republic of Moldova; 10 National Agency for Public Health, Chisinau, Republic of Moldova

## Abstract

The Republic of Moldova faces several concurrent health challenges most notably an increase in chronic non-communicable diseases, spiralling health care costs and widening health inequalities. To accelerate progress in their resolution there is a need for new and innovative health promotion and behaviour change communication interventions. The Ministry of Health, Labour and Social Protection in collaboration with the newly created National Agency for Public Health held a conference on the occasion of the *Moldovan National Day of Health Promotion* on 14th March 2018 in which national and invited international experts exchanged their views on (1) best practice examples of behaviour change interventions, health promotion activities and lessons learned from the UK and elsewhere; and (2) possible ways forward for Moldova to implement cost-effective and evidence-based intersectoral health promotion programmes. The experts provided recommendations on implementing behaviour change interventions to reduce and prevent obesity; on the creation of a favourable tobacco control environment to reduce smoking prevalence; and on how physical activity programme design can benefit from health psychology research. All these strategies could foster health promotion activities and ultimately contribute to improving the health outcomes of the Moldovan population.

## Background

### Epidemiological transition and non-communicable diseases in Moldova

Non-communicable diseases (NCDs) accounted for about 70% of all deaths globally in 2015 and more than 80% of these premature deaths were in low-income and middle-income countries (LMICs) [1]. Like many countries, the Republic of Moldova (hereinafter Moldova) faces a recent transition in the profile of health affecting the health status of its population with a growing NCD burden [2]. The prevalence of NCDs in the country is very high, accounting for 90% of all-cause mortality in 2016. The major NCDs are cardiovascular diseases (CVDs) and cancers, accounting for 59 and 15% of all deaths, respectively [3].

Alcohol consumption and tobacco smoking are the key health risks for most Moldovans and mortality and morbidity from these risk factors account for a sizeable disease burden on society [3]. According to 2010 figures, 58% of total male mortality and 62% of female mortality could be attributed to smoking-related causes, while 19% of male mortality and 14% of female mortality were related to alcohol consumption [4, 5]. There is an uneven distribution of the major risk factors with men having higher rates of engaging in tobacco smoking and alcohol consumption (Table 1) [3, 6, 7]. As shown in Table 1, overweight and obesity constitutes another major public health problem in Moldova with 47% of its adult population (18 and over) being classified as overweight or obese (BMI ≥ 25) in 2014 [7]. Current physical activity levels in Moldova are unclear [8], with 2013 data suggesting 10% of the population is insufficiently active while 76% does not engage in vigorous activity [9]. Physical inactivity however is one of 10 leading behavioural risk factors contributing to disease burden in Moldova [8]. High systolic blood pressure is the second contributor to burden of disease in Moldova after dietary risks [8].

**Table 1:** Major risk factors among the Moldovan population

*Risk factors*	Women [%]	Men [%]	Total [%]
Overweight (2014)	46.7	46.4	46.6
Tobacco smoking (2013)	5.4	44.8	23.8
Alcohol consumption (2010)	8.9	25.9	16.8
Raised blood pressure (2008)	39.6	41.3	40.4

However, these estimates show that there is a lack of more recent, aggregated, data on the burden of NCDs and associated risk factors amongst the Moldovan population [2, 3, 6, 7]. The existing system for NCD surveillance is not operational, and data on risk factors and behaviours related to NCDs are not routinely collected as part of the country’s health information system [3].

### NCD and health promotion policy environment in Moldova

The Government of Moldova has embarked on a health system reform to primarily strengthen the delivery and quality of primary health care since the late 1990s [4]. Moldova made considerable advances in reorienting its hospital-focused health system towards primary healthcare (PHC) based on a family medicine model where private health providers are directly contracted by the health insurance system [4]. The health insurance, however, inadequately covers costs for both the provider and the patient with high out-of-pocket costs for medicines, and thus limits patients’ access to critical therapies and care, especially for chronic illnesses [2, 10]. Moreover, there are few state-supported health promotion activities sustained by the PHC system and family doctors continue to be largely focused on the provision of curative services, whilst public health promotion and prevention activities are considered to be of a lesser concern in resource-constrained circumstances. This is also exemplified by the proportion of public health expenditures from 2010, which is high for curative services (68%) and very low for public health and prevention (5%) [4].

The Ministry of Health, Labour and Social Protection (MoHLSP) has made progress in establishing an extensive legal and policy framework relevant to the control and improvement of service delivery for NCDs. NCDs have gained high priority in two overarching national policy frameworks for health; the Moldovan National Health Policy for 2007–2021 [11], and the Health Care System Development Strategy for 2008–2017 [12]. Both pursue the goal of significantly reducing the NCD disease burden and avoiding premature deaths through integrated actions, improving the quality of life and healthy life expectancy [2]. In line with national policy commitments, the Parliament has approved a NCD Prevention and Control Strategy 2012–2020 in 2012 [13]. The core focus of this strategy is the control of the main NCDs and their risk factors, as well as opportunities to prevent them. At the same time, it has launched several national programmes related to alcohol and tobacco control to address major risk factors for NCDs [2]. A national Health Promotion Programme encourages healthy behaviours at all stages of life through the modification of attitudes and improvement of knowledge among adults, adolescents and youth. Capacity building of health and education specialists on planning and implementing health promotion actions at national and local level are at the core of this Programme. However, even though NCDs gained political momentum, progress in *implementing* national chronic disease programmes and strategies has been slow with limited financial resources for implementation [2]. The burden of NCDs and related health care costs still undermine social and economic development in Moldova and represent an urgent public health priority [14].

### The healthy life project “Reducing the Burden of NCDs in Moldova”

The Swiss Agency for Development and Cooperation’s (SDC) Healthy Life Project was launched in 2017 to support the MoHLSP in reducing the burden of NCDs in Moldova as part of a bilateral development aid agreement. The Healthy Life Project is implemented by the Swiss Tropical and Public Health Institute (Swiss TPH) and its national and local partners, i.e. the raion (district) and local public authorities, medical teaching and training institutes for family health, primary health care and public health services, and community-based organisations. The project is designed to support the development of an enabling policy environment, to improve the quality uptake of NCD services at PHC level, and to increase the healthcare seeking behaviour of the population [15].

On the occasion of the Moldovan National Day of Health Promotion specialist on 14th March 2018, a two-day working conference was organised by the MoHLSP and the newly created National Agency for Public Health in Chisinau. The aim of this conference was to share the latest evidence in health promotion and behaviour change for NCDs by international and national experts in an interactive workshop setting. The objective of the workshop was to develop action plans for behaviour change interventions by multisectoral raion teams, taking into account latest scientific evidence and international best practice examples. To this end, the conference organisers invited international health promotion, behaviour change and health psychology experts from their professional networks, national public health policy experts and practitioners, representatives from medical, social, educational sectors, community leaders and local public authorities as well as the representatives from the MoHLSP to participate as speakers and panellists. As the conference aimed at many non-English speaking health professionals facing a language barrier in their access to research literature, the conference was being translated into Romanian and Russian in real time alongside the slides. As an outcome of the conference, this paper specifically describes:

1. best practice examples of behaviour change interventions, health promotion activities and lessons learned from the UK on the topics of physical activity, obesity and tobacco cessation (explained by three international conference speakers)
2. recommendations for Moldova on how to adapt some of these health promotion strategies to its context to improve the health status of the Moldovan population.

We begin our summary of the conference with three best practice examples of health promotion and behaviour change interventions in the U.K. in the areas of overweight and obesity guidelines development, tobacco cessation and physical activity. We will then highlight recommendations from the speakers for guiding the development of behaviour change strategies in Moldova adapted to the national context and resources for a successful promotion of healthy lifestyles.

### Case study 1: developing national guidance for effective diet and obesity interventions

Overweight and obesity are increasingly affecting the population of the UK; the prevalence of obesity rose from 6% of men and 8% of women in 1980 to 26% of men and 27% of women in 2016 [16, 17]. Overweight is more common than obesity among the UK population, with 40% of men and 30% of women classified as overweight in 2016 [17]. The illnesses related to an excess of weight include type 2 diabetes, hypertension, stroke, dyslipidaemia, osteoarthritis and some cancers [18, 19]. The treatment of obesity remains challenging and requires multicomponent weight management programmes, but up to 2014, existing service provision was often limited in scope [20, 21].

It is now widely recognised that multiple interventions at multiple levels are often needed to initiate and sustain behaviour change effectively. Interventions on social and behavioural factors related to health should link multiple levels of influence, including the individual, interpersonal, institutional, community, and policy levels [22]. Public health interventions should not only be targeted at individuals, but also affect interpersonal, organisational, and environmental factors influencing health behaviour as it is increasingly recognised that individual behaviour change affects and is affected by the larger socioecological environment [23, 24]. There is increasing emphasis on identifying evidence-based interventions and disseminating them widely [25]. One of these examples is the development of evidence-based national guidance and advice by the National Institute for Health and Care Excellence (NICE).

### Development of a national guidance on overweight and obesity prevention and management

Since 1999 NICE has been developing recommendations on public health interventions in the UK using the best available evidence for disease prevention and control for those working in health and social care in England [26]. The role of NICE is to improve outcomes for people using the National Health Service (NHS) and other public health and social care services. NICE pursues an evidence-based approach to produce guidance and advice for health, public health, and social care practitioners. It has produced a number of guidelines on diet management, behaviour change and physical activity that make recommendations on local interventions to help prevent disease or improve health [26], such as its guidance on managing overweight and obesity.

In 2014, NICE has up-dated its evidence-based guidance on obesity and weight management [27], obesity prevention [27], as well as on lifestyle services for overweight or obese adults [28]. As summarised in Table 2, this guidance outlines how public health services and local authorities can increase physical activity levels in a variety of settings (early childhood development settings, schools and workplaces) and achieve dietary changes among their target populations. It covers the core component for effective lifestyle weight management services and recommends an integrated approach to preventing and managing obesity. Moreover, it also addresses community-wide strategies to prevent obesity through the sustainable engagement of local communities, local organisations, and networks.

**Table 2:** Overview of behaviour change interventions covered by the different guidance on obesity

Guidance	Title	Interventions														
		Behaviour change interventions	Advice and referral – physical activity	Advice and referral - diet	Lifestyle weight management programmes	Workplace interventions	School based interventions	Community based interventions	Self-help	Media	Training, communication and professional skills	Local education initiatives	Promoting Breastfeeding	Cultural appropriateness	Partnership working and JSNA	Planning, monitoring and evaluation
NG7	Preventing excess weight gain	**Y**	**Y**	**Y**	**Y**	**N**	**N**	**N**	**N**	**N**	**N**	**N**	**N**	**Y**	**Y**	**N**
PH53	Weight management: lifestyle services for overweight or obese adults	**Y**	**Y**	**Y**	**Y**	**N**	**N**	**Y**	**Y**	**N**	**Y**	**N**	**N**	**N**	**Y**	**Y**
PH47	Weight management: lifestyle services for overweight or obese children and young people	**Y**	**Y**	**Y**	**Y**	**N**	**N**	**Y**	**Y**	**Y**	**Y**	**N**	**N**	**N**	**Y**	**Y**
PH46	BMI: preventing ill health and premature death in black, Asian and other minority ethnic groups	**Y**	**Y**	**Y**	**N**	**N**	**N**	**Y**	**Y**	**Y**	**N**	**Y**	**N**	**N**	**N**	**N**
PH42	Obesity: working with local communities	**Y**	**N**	**N**	**N**	**Y**	**N**	**Y**	**N**	**N**	**Y**	**Y**	**Y**	**Y**	**Y**	**Y**
PH27	Weight management before, during and after pregnancy	**Y**	**Y**	**Y**	**N**	**N**	**N**	**Y**	**Y**	**N**	**Y**	**Y**	**Y**	**N**	**N**	**N**
CG43	Obesity prevention	**Y**	**Y**	**Y**	**N**	**Y**	**Y**	**Y**	**N**	**N**	**Y**	**N**	**N**	**N**	**Y**	**N**

Examples of how the guidance has been put into practice and evaluated in the NHS, local authorities, voluntary sector and a range of other organisations can be found on the NICE shared learning pages [29, 30].

### Case study 2: effective behaviour change interventions for smoking cessation and prevention

Smoking remains the largest, preventable cause of death worldwide, killing more than 7 million people around the world each year, of them 114,513 per year in the UK [31]. Tobacco use is highly addictive, resulting in only half of smokers managing to quit tobacco completely in their lifetime [32]. Not only does smoking have dire health consequences - smokers die on average at least 10 years earlier than non-smokers [33, 34] - but it also causes high health costs and loss of productivity [35]. Reducing tobacco consumption is therefore likely not only going to improve the health but also the wealth of a nation [36].

There are several frameworks that help in the development, evaluation and categorisation of tobacco control interventions. One of them is the conceptualisation of tobacco control methods into their mode of action and level of delivery which work at different stages of the tobacco epidemic [37].

At the earliest stage of the epidemic, increasing awareness in the population of the consequences of tobacco use through mass media campaigns showed positive results [38]. At the same time, publicising basic research likely increases awareness and can change behaviour not only of smokers themselves but also of health professionals to support tobacco control [39]. Lastly, changing social norms and values can create a favourable atmosphere for tobacco control [40].

Taxation is one of the most effective ways to reduce tobacco consumption and is often introduced at later stages in the epidemic [41]. The introduction of clean air laws and smoking bans can have a similar impact on tobacco use as tax increases [42]. Finally, product regulation can help make cigarettes less addictive and therefore less attractive and can help encourage smokers to switch to less dangerous products to reduce harm [43, 44].

At the latter stages of the tobacco epidemic, intervention programmes operating at the individual level tend to be implemented. They usually mobilise a combination of approaches ranging from behavioural support interventions (such as brief counselling, telephone counselling or intensive face-to-face support) to e-health approaches (text messaging, internet and apps) and pharmacotherapy (nicotine replacement therapy and Varenicline). Table 3 further elaborates on the range of effective behaviour change interventions for tobacco cessation and prevention which are available for policy-makers and intervention designers.

**Table 3:** Overview of behaviour change interventions for tobacco cessation and prevention

Intervention domains for smoking cessation and prevention		
Behavioural Support	M/E-Health	Pharmacotherapy
***Brief counselling*** • Usually provided by a health professional • Involves asking patients about their smoking status, advising them to quit and assisting with further help (e.g. prescription or referral). • Takes no more than 5 min and can increase long-term abstinence rates by 1–3% [45]. ***Telephone counselling*** • Proactive and lasts at least three sessions • Involves motivational messages, advice for self-regulation,self-monitoring and social support • Can increase long-term abstinence rates by 2–4% [45]. ***Intensive face-to-face support*** • Provided one-to-one or in groups and usually lasts for at least 4-6 weekly sessions • Involves provision of social support, advice, and encouragement to minimise motivation to smoke and maximise motivation to remain abstinent, increase the skills and capacity for self-control and optimise effective medication use. • Strengthening ex-smoker identity, providing rewards and advising on changing routine have proven to be particularly important [46]. • Can increase long-term abstinence rates by 10% [47, 48].	***Text messaging*** • Wide-reaching and cheap way to communicate with smokers • Involves the sending of targeted messages to motivate smokers to remain abstinent, provide information and advice on self-regulation. • Can increases abstinence by up to 5% [49, 50] ***Internet/Apps*** • Can reach a large number of smokers, provide information in graphic form, have the option for interactive feedback and can exploit economies of scale. • Evidence for internet-based interventions and apps is currently limited by the poor quality of most studies [51, 52].	***Nicotine replacement therapy*** • Includes various different forms (including gum, patch and spray) • Should be provided for at least 8 weeks. • Decreases the motivation to smoke by reducing cravings and withdrawal symptoms, reducing the rewarding effect of smoking and providing behavioural control. • Can Increase long-term abstinence rates by around 10% [53, 54]. ***Varenicline*** • Partial nicotine receptor • Should be taken for at least 12 weeks • Reduces cravings and withdrawal symptoms and the rewarding effects of smoking • Is the most effective pharmacotherapy available [55], can increase long-term abstinence rates by around 15% [56]. ***Combination therapy*** • A combination of pharmacotherapy with behavioural support provides the best long-term outcomes for smoking cessation; can lead to abstinence rates of around 20% at 12 months [57]. • Evidence available that a combination of different pharmacotherapy is more effective than single form pharmacotherapy [58, 59].

In general, successful behaviour change interventions for smoking cessation and prevention are among the cost-effective healthcare interventions [60, 61]. However, there is a trade-off insofar as cheaper, less intensive interventions have a lower impact than more expensive, intensive interventions but the latter may be less affordable for LMICs [62]. Overall, it is the combination of pharmacotherapy with behavioural support which provides the best long-term outcomes for smoking cessation.

### Case study 3: designing an effective community-based physical activity programme for those with CVD risk and poor mental health

Physical activity is one of the most underused behavioural medicines, with 40% of adults in the UK [63], 45% in Australia [64], and 79% in America [65], failing to reach the World Health Organization (WHO) recommendations of 150 min of moderate to vigorous physical activity per week [66]. In addition to these recommendations, it is important to break up sitting time in order to reduce the risk of non-communicable disease [67]. Physical inactivity is the fourth leading risk factor for mortality, accounting for 6% of deaths worldwide [66]. Hence, the practise of regular physical activity has huge potential to reduce all-cause mortality and enhance good health [68, 69], protecting against and benefiting the treatment of CVDs [70], stroke, cancer, type 2 diabetes [71], and depression [72]. Public health programmes that aim to enhance physical activity are therefore of priority.

In order to enable a global understanding of what works to enhance physical activity and target NCDs, it is argued that: 1) behavioural science and public health must work together and 2) effective interventions are not developed ‘*by chance’*, instead the intervention design, delivery, evaluation and adoption must draw upon relevant strategies, frameworks and approaches to perform and address a systematic behavioural diagnosis of the target population [73–75].

The community-based health programme ‘Active Herts’ aimed to enhance physical activity, health and wellbeing in those with CVD risk and poor mental health [76]. It was informed by the Behaviour Change Wheel (BCW) and COM-B model of behaviour [77], and the latest evidence from health psychology using this model to explain physical activity [78]. Table 4 provides an overview of some of the core determinants that were targeted based on a COM-B behavioural diagnosis.

**Table 4:** COM-B diagnosis of factors that influence participation in physical activity

Capability (Psychological and Physical)	Opportunity (Physical and Social)	Motivation (Reflective and Automatic)
Unaware of guidelines in relation to own behaviour	No-one to go with	Lack of confidence
Often forgets to be active	Lack of support to be active	Low outcome expectancies
Lack of headspace to plan	Not enough money for gym	Habitual inactivity
Low stamina/energy	Unsupportive environment	Restricted by emotion (nervous/scared)

*COM-B Capability, Opportunity and Motivation

To ensure scientific rigour and long-term effectiveness, the programme drew from a systematic review of the literature that highlighted successful strategies for physical activity enhancement and/or sedentary behaviour reduction [79, 80]. Table 5 shows the behaviour change techniques (BCTs) drawn from the 93 item taxonomy that were found to be effective at changing physical activity behaviour from the review of published evidence [81].

**Table 5:** Behaviour change techniques (BCTs) frequently found to be applied in effective physical activity interventions

Behaviour change techniques	Explanation/definition
Biofeedback	Provide feedback about the body using an external device [78].
Demonstration of the behaviour	Provide an observable example of the performance of the behaviour for imitation [78].
Behaviour practice/rehearsal	Prompt practice or rehearsal of a behaviour in order to increase habit and skill [78].
Graded tasks	Set easy-to-perform tasks, making them progressively difficult but achievable until behaviour is performed [78].
Action planning	Prompt a detailed planning of how/where/when to perform the behaviour [78].
Instruction on how to perform the behaviour	Advise on how to perform the behaviour [78].
Prompts/cues	Set environmental or social stimulus with the purpose of prompting the behaviour [78].
Self-reward	Prompt self-praise or self-reward if there has been progress towards performing the behavior [78].

Based on these findings, the ‘Active Herts’ programme was designed to include the BCTs shown to be the most effective at enhancing physical activity, alongside others that target Capability, Opportunity and Motivation (COM-B). The programme consisted of a 45 min one-to-one consultation and a phone call booster (2 weeks later) with a Registered Exercise Professional (Get Active Specialist: GAS) who had been specifically trained in behaviour change, motivational interviewing and health coaching [75, 82]. The GAS supported programme users through the consultation, using an Active Herts booklet (focusing on BCTs such as *goal setting, problem solving, action planning, prompts/cues, self-reward, social support*), and then referred them to local exercise classes (*instruction on how to perform the behaviour, demonstration of the behaviour, behaviour practice/rehearsal*), that were free in two of the four programme areas. To support replicability, a protocol was published and made open access [76]. Early evaluation results show that there was a significant increase in the number of programme users who reported engaging in regular physical activity 12 months after the intervention, alongside improved health and wellbeing levels [75].

Physical activity bears great potential for maintaining health and treating illness. Applying behavioural science in public health settings to understand and change behaviour can maximise the scale of the impact of public health interventions and assist in the mission to improve world-wide population health and wellbeing.

## Discussions and recommendations for Moldova – adopting evidence-based approaches to health promotion

The final session of the conference was dedicated to discussing and identifying potential strategies to effectively implement behaviour change interventions and health promotion activities within primary health care settings at the raion level in Moldova. The following key recommendations resulted from this discussion and the experts interventions.

### Prioritize cost-effective and evidence-based interventions to reduce and prevent obesity

Moldova should consider implementing cost-effective and evidence-based interventions to reduce the high prevalence of obesity. Prevention of obesity through lifestyle weight management programmes delivered by skilled providers is an option. NICE provides recommendations on the core components for effective weight loss and Moldova should consider implementing some of these measures in a coordinated way through multidisciplinary providers [81]. Given the limited resources available within Moldova, when considering implementing behaviour change interventions to reduce obesity it is worthwhile to remember that potential additional costs may be incurred when training staff, including those not directly involved in services providing behaviour change interventions. Costs can also be incurred when evaluating and monitoring behaviour change interventions. However, interventions aimed at changing people’s health-related behaviours have the potential to improve their health and wellbeing considerably and any costs can be offset by disinvesting in interventions where evidence shows them to be not effective or harmful. These savings could be reinvested in effective evidence-based services.

### Create a favourable tobacco control environment to reduce smoking prevalence

Moldova should consider increased taxation for tobacco products to drive down smoking prevalence. Existing social norms around gender-specific smoking rates should be exploited to create a favourable tobacco control environment. Further mass media campaigns may be useful, but only if there is evidence of low awareness of the consequences of tobacco use in the general population.

Primary physicians have a key role in helping people to stop smoking through brief counselling (see Table 3). Increasing the number of General Practitioners providing smoking cessation advice is likely to have an impact at population level and is cheap (around US$19). These cost estimates were derived from the Affordability Calculator [83], whereby Moldova specific data on GDP were used with additional reference data based on Russian estimates. Furthermore, the rise in the popularity of e-cigarettes has provided the opportunity for a consumer-driven intervention which may reduce smoking rates via harm reduction [83]. E-cigarettes can become cheaper than cigarettes (around US$20–25) and as this approach is self-funded it would be free to governments. Nicotine replacement therapy with cytisine which is similar in action to varenicline can be just as effective but is much cheaper (US$20) [62, 77, 84]. The Law on tobacco control was amended by the Parliament of Moldova in July 2019 towards increased regulation of non-burning tobacco products, in line with the position of WHO recognizing that electronic nicotine delivery systems (ENDS) are not harmless and the evidence of the impact on health remains limited [85]. Whether or not e-cigarettes should be used in the framework of a harm reduction approach is currently debated [86, 87].

Furthermore, text-based interventions are cheap (around US$39) and can have a wide reach (see Table 3). They are therefore ideal in the context of Moldova. However, the usefulness of text-based interventions partly depends on the extent of skilled mobile phone use in specific population groups such as the elderly, and the quality of existing networks, especially in remote areas.

Similarly, tailored self-help material is cheap (around US$10–14) and has similar effectiveness to brief advice [88]. This is also true for telephone quit lines which are cost-effective and can reach large sways of the population. Lastly, while more evidence needs to accumulate for internet and app-based smoking cessation interventions, their wide reach and low cost makes them an ideal candidate for inclusion in comprehensive treatment packages for LMICs such as Moldova. Because developments in this area are rapid, these tools should be closely monitored as evidence is increasing to support their effectiveness, and they may be particularly suitable for targeting younger or more disadvantaged smokers [89, 90].

### Design and implement evidence-based, community programmes on physical activity

In Moldova there is a need to develop and maintain national guidelines and surveillance for levels of physical activity in the general population [91]. This is essential to assess current needs for increased intervention and design adapted behaviour change programmes to support a beneficial change in behaviour. Furthermore, physical activity intervention or programme developers should consider evidence-based frameworks, theoretical approaches and insights from health psychology and behavioural science to guide intervention design, development, delivery, evaluation and adoption strategies (IDDEAS: [92, 93]). Community-based programmes jointly implemented by skilled providers across all sectors, with targeted individual behavioural support, should be the preferred level of intervention, especially in lower-income settings to avoid a waste in resource. A detailed protocol developed that explains what the intervention aims to do, how it will do it and why can aid in replication and roll-out if the programme is successful. Following the Active Herts protocol could support the development of future interventions in this area using the evidence-based and theoretically-driven behaviour change techniques and approaches mentioned above [76] and further research into local health behaviour determinants in targeted population groups in Moldova are encouraged to use this approach.

## Conclusions

The case studies shown during the Conference described here provide a number of best practice interventions adaptable to Moldova, including options for resource-constrained settings. Health psychology research can inform the development of appropriate individual and population health promoting strategies but access by Moldovan specialists to this body of scientific evidence requires sustained support and investment. Overall, there is growing awareness of the complexities of embedding behavioural science in public health practice more widely [94]. It will be important that health promotion strategy development benefit from extensive and appropriately funded stakeholder consultations to ensure the inclusion of the latest evidence.

The implementation of cost-effective and evidence-based interventions to reduce obesity, the creation of a favourable tobacco control environment to reduce the smoking prevalence and the design of community-based programmes for physical activity is expected to foster national behaviour change communication and health promotion activities and in a longer-term, contribute to strengthening the national health system and improve the health outcomes of the Moldovan population [95, 96].

## Data Availability

Not applicable.

## Abbreviations

BCTs: Behaviour change techniques
BCW: Behaviour change wheel
BMI: Body mass index
COM-B: Capability, opportunity and motivation
CVDs: Cardiovascular diseases
ENDS: Electronic nicotine delivery systems
GAS: Get Active Specialist
IDDEAS: Intervention design, development, delivery, evaluation and adoption strategies
JSNA: Joint strategic needs assessment
LMICs: Low-income and middle-income countries
MoHLSP: Ministry of health, labour and social protection
NCDs: Non-communicable diseases
NHS: National health service
NICE: National Institute for Health and Care Excellence
PHC: Primary healthcare
SDC: Swiss Agency for Development and Cooperation
Swiss TPH: Swiss Tropical and Public Health Institute
UK: United Kingdom
WHO: World Health Organization

## References

[1] WHO: Global Health Observatory Data. NCD mortality and morbidity. [http://www.who.int/gho/ncd/mortality_morbidity/en/], accessed: [April 10, 2019].

[2] Skarphedinsdottir M, Smith B, Ferrario A, Zues O, Ciobanu A, Tirdea M, Domente S, Habicht J (2014). Better noncommunicable disease outcomes: challenges and opportunities for health systems: Republic of Moldova country assessment.

[3] WHO. Noncommunicable diseases country profiles. Republic of Moldova; 2018. [https://www.who.int/nmh/countries/mda_en.pdf?ua=1], accessed: [April 10, 2019]

[4] Turcanu G, Domente S, Buga M, Richardson E (2012). Republic of Moldova: health system review. Health Syst Transit.

[5] WHO (2012). Evaluation of the structure and provision of primary care in the Republic of Moldova.

[6] Ministry of Health. National Strategy on Public Health for years 2014-2020, approved by the Governmental Decision nr. 1032 from 20 December 2013 and published in Monitorul Oficial N 304-310 on 27 December 2013.

[7] WHO: Republic of Moldova (2016). Highlights on health and well-being.

[8] WHO: Republic of Moldova: profile of health and well-being. [http://www.euro.who.int/__data/assets/pdf_file/0005/323258/Profile-health-well-being-Rep-Moldova.pdf?ua=1], accessed: [April 10, 2019].

[9] WHO (2014). Prevalence of non-communicable disease risk factors in the Republic of Moldova - STEPS 2013.

[10] Vian T, Feeley FG, Domente S, Negruta A, Matei A, Habicht J (2015). Barriers to universal health coverage in Republic of Moldova: a policy analysis of formal and informal out-of-pocket payments. BMC Health Serv Res.

[11] Government of the Republic of Moldova: Decision no 886 from 06.08.2007 regarding Approval of the National Health Policy Monitorul Oficial no 127–130, art. 931 [http://lex.justice.md/md/324940/], accessed: [April 10, 2019].

[12] Government of the Republic of Moldova: Decision no 1471 from 24.12.2007 regarding Approval of the National Healthcare Strategy for the years 2008–2017. Monitorul Oficial 8–10, art 43. [http://lex.justice.md/md/326615/], accessed: [April 10, 2019].

[13] Parliament of the Republic of Moldova: Decision no 182 from 12.04.2012 regarding Approval of National Strategy of Prevention and Control of Non-Communicable Diseases for the years 2012–2020. Monitorul Oficial no 126–129, art 412. [http://lex.justice.md/viewdoc.php?action=view&view=doc&id=343682&lang=1], accessed: [April 10, 2019].

[14] Hone T, Habicht J, Domente S, Atun R (2016). Expansion of health insurance in Moldova and associated improvements in access and reductions in direct payments. J Glob Health.

[15] Swiss TPH Healthy Life Project - Reducing the burden of non-communicable diseases in Moldova. [https://www.swisstph.ch/en/projects/project-detail/project/healthy-life-project-reducing-the-burden-of-non-communicable-diseases-in-moldova/], accessed: [April 10, 2019].

[16] Rennie KL, Jebb SA (2005). Prevalence of obesity in Great Britain. Obes Rev.

[17] Health and Social Care Information Centre (2016). Health survey for England.

[18] Burton B, Foster W, Hirsch J, Van TI (1985). Health implications of obesity: an NIH consensus development conference. Int J Obes.

[19] Must A, Spadano J, Coakley EH, Field AE, Colditz G, Dietz WH (1999). The disease burden associated with overweight and obesity. Jama.

[20] NHS England: Joined up clinical pathways for obesity: Report of the working group. [https://www.england.nhs.uk/wp-content/uploads/2014/03/owg-join-clinc-path.pdf], accessed: [April 10, 2019].

[21] Stegenga H, Haines A, Jones K, Wilding J (2014). Identification, assessment, and management of overweight and obesity: summary of updated NICE guidance. BMJ.

[22] Syme SL, Smedley BD (2000). Promoting health: intervention strategies from social and behavioral research.

[23] NICE (2007). Behaviour change at population, community and individual levels [NICE public health guidance 6].

[24] Davis R, Campbell R, Hildon Z, Hobbs L, Michie S (2015). Theories of behaviour and behaviour change across the social and behavioural sciences: a scoping review. Health Psychol Rev.

[25] Glanz K, Rimer BK, Viswanath K (2008). Health behavior and health education: theory, research, and practice.

[26] NICE: What we do. [https://www.nice.org.uk/about/what-we-do], accessed: [April 10, 2019].

[27] NICE: Obesity: identification, assessment and management. Clinical guideline [CG189]. [https://www.nice.org.uk/guidance/cg43], accessed: [April 10, 2019].

[28] NICE: Obesity prevention. Clinical guideline [CG43]. [https://www.nice.org.uk/guidance/cg43], accessed: [April 10, 2019].

[29] NICE: Choose to change service delivered by ABL Health (ABL): Adults about to complete a lifestyle weight management programme agree a plan to prevent weight regain. [https://www.nice.org.uk/sharedlearning/choose-to-change-service-delivered-by-abl-health-abl-adults-about-to-complete-a-lifestyle-weight-management-programme-agree-a-plan-to-prevent-weight-regain], accessed: [April 10, 2019].

[30] NICE: 2018 Surveillance of obesity: identification, assessment and management (2014) NICE guideline CG189. [https://www.nice.org.uk/guidance/cg189/evidence/appendix-a2-summary-of-evidence-from-surveillance-cg189-pdf-4847559663], accessed: [April 10, 2019].

[31] World Lung Foundation/American Cancer Society: The tobacco atlas. [http://www.tobaccoatlas.org], accessed: [April 10, 2019].

[32] WHO (1997). Tobacco or health: a global status report.

[33] Doll R, Peto R, Boreham J, Sutherland I (2004). Mortality in relation to smoking: 50 years’ observations on male British doctors. BMJ.

[34] Pirie K, Peto R, Reeves GK, Green J, Beral V, Million Women Study C (2013). The 21st century hazards of smoking and benefits of stopping: a prospective study of one million women in the UK. Lancet.

[35] Lightwood J, Collins D, Lapsley H, Novotny TE, Jha P, Chaloupka F (2000). Estimating the cost of tobacco use, in Tobacco control in developing countries.

[36] Jha P, Chaloupka FJ (1999). Curbing the epidemic: governments and the economics of tobacco control.

[37] Slama K, Control T, Sancho-Garnier H (2004). Evidence-based cancer prevention: strategies for NGOs.

[38] Hopkins DP, Briss PA, Ricard CJ, Husten CG, Carande-Kulis VG, Fielding JE, Alao MO, McKenna JW, Sharp DJ, Harris JR, Woollery TA, Harris KW (2001). Reviews of evidence regarding interventions to reduce tobacco use and exposure to environmental tobacco smoke. Am J Prev Med.

[39] Lundberg GD (1985). In the AMA, policy follows science: a case history of tobacco. JAMA.

[40] Aquilino ML, Lowe JB (2004). Approaches to tobacco control: the evidence base. Eur J Dent Educ.

[41] Ranson MK, Jha P, Chaloupka FJ, Nguyen SN (2002). Global and regional estimates of the effectiveness and cost-effectiveness of price increases and other tobacco control policies. Nicotine Tob Res.

[42] Stephens T, Pederson LL, Koval JJ, Kim C (1997). The relationship of cigarette prices and no-smoking bylaws to the prevalence of smoking in Canada. Am J Public Health.

[43] Henningfield JE, Benowitz NL, Slade J, Houston TP, Davis RM, Deitchman SD (1998). Reducing the addictiveness of cigarettes. Council on scientific affairs, American medical association. Tob Control.

[44] Stratton S, Shetty P, Wallace R, Bondurant S (2001). Clearing the smoke: addressing the science base for tobacco harm reduction.

[45] Stead LF, Buitrago D, Preciado N, Sanchez G, Hartmann-Boyce J, Lancaster T (2013). Physician advice for smoking cessation. Cochrane Database Syst Rev.

[46] West R, Walia A, Hyder N, Shahab L, Michie S (2010). Behavior change techniques used by the English stop smoking services and their associations with short-term quit outcomes. Nicotine Tob Res.

[47] Lancaster T, Stead LF (2017). Individual behavioural counselling for smoking cessation. Cochrane Database Syst Rev.

[48] Stead LF, Lancaster T (2005). Group behaviour therapy programmes for smoking cessation. Cochrane Database Syst Rev.

[49] Free C, Knight R, Robertson S, Whittaker R, Edwards P, Zhou W, Rodgers A, Cairns J, Kenward MG, Roberts I (2011). Smoking cessation support delivered via mobile phone text messaging (txt2stop): a single-blind, randomised trial. Lancet.

[50] Whittaker R, McRobbie H, Bullen C, Borland R, Rodgers A, Gu Y (2012). Mobile phone-based interventions for smoking cessation. Cochrane Database Syst Rev.

[51] Shahab L, McEwen A (2009). Online support for smoking cessation: a systematic review of the literature. Addiction.

[52] Haskins BL, Lesperance D, Gibbons P, Boudreaux ED (2017). A systematic review of smartphone applications for smoking cessation. Transl Behav Med.

[53] West R, Shahab L, Killoran A, Kelly M (2010). Smoking cessation interventions, in evidence-based public health.

[54] Stead LF, Perera R, Bullen C, Mant D, Hartmann-Boyce J, Cahill K, Lancaster T (2012). Nicotine replacement therapy for smoking cessation. Cochrane Database SystRev.

[55] Anthenelli RM, Benowitz NL, West R, St Aubin L, McRae T, Lawrence D, Ascher J, Russ C, Krishen A, Evins AE (2016). Neuropsychiatric safety and efficacy of varenicline, bupropion, and nicotine patch in smokers with and without psychiatric disorders (EAGLES): a double-blind, randomised, placebo-controlled clinical trial. Lancet.

[56] Cahill K, Lindson-Hawley N, Thomas KH, Fanshawe TR, Lancaster T (2016). Nicotine receptor partial agonists for smoking cessation. Cochrane Database Syst Rev.

[57] Ferguson J, Bauld L, Chesterman J, Judge K (2005). The English smoking treatment services: one-year outcomes. Addiction.

[58] Cahill K, Stevens S, Perera R, Lancaster T (2013). Pharmacological interventions for smoking cessation: an overview and network meta-analysis. Cochrane Database Syst Rev.

[59] Chang PH, Chiang CH, Ho WC, Wu PZ, Tsai JS, Guo FR (2015). Combination therapy of varenicline with nicotine replacement therapy is better than varenicline alone: a systematic review and meta-analysis of randomized controlled trials. BMC Public Health.

[60] Shahab L (2011). NCSCT briefing 7: cost-effectiveness of pharmacotherapy for smoking cessation.

[61] Shahab L (2015). NCSCT briefing: effectiveness and cost-effectiveness of programmes to help smokers to stop and prevent smoking uptake at local level.

[62] West R, Raw M, McNeill A, Stead L, Aveyard P, Bitton J, Stapleton J, McRobbie H, Pokhrel S, Lester-George A, Borland R (2015). Health-care interventions to promote and assist tobacco cessation: a review of efficacy, effectiveness and affordability for use in national guideline development. Addiction.

[63] Her Majesty's Government Department for Digital C, Media & Sport, : Sporting future: first annual report. [https://assets.publishing.service.gov.uk/government/uploads/system/uploads/attachment_data/file/590578/Sporting_Future_-_first_annual_report_final.pdf], accessed: [April 10, 2019].

[64] Australian Government Department of Health: The national health survey 2014–2015. [http://www.health.gov.au/internet/main/publishing.nsf/content/health-pubhlth-strateg-active-evidence.htm], accessed: [April 11, 2019].

[65] Centers for Disease Control and Prevention: Facts about physical activity. [https://www.cdc.gov/physicalactivity/data/facts.htm], accessed: [April 11, 2019].

[66] World Health Organization: Global recommendations on physical activity for health: 18–64 year olds. [http://www.who.int/dietphysicalactivity/physical-activity-recommendations-18-64years.pdf?ua=1], accessed: [April 11, 2019].

[67] Bailey DP, Locke CD (2015). Breaking up prolonged sitting with light-intensity walking improves postprandial glycemia, but breaking up sitting with standing does not. J Sci Med Sport.

[68] Bauman AE (2004). Updating the evidence that physical activity is good for health: an epidemiological review 2000-2003. J Sci Med Sport.

[69] Lollgen H, Bockenhoff A, Knapp G (2009). Physical activity and all-cause mortality: an updated meta-analysis with different intensity categories. Int J Sports Med.

[70] Barengo NC, Antikainen R, Borodulin K, Harald K, Jousilahti P (2017). Leisure-time physical activity reduces total and cardiovascular mortality and cardiovascular disease incidence in older adults. J Am Geriatr Soc.

[71] Rhodes RE, Janssen I, Bredin SSD, Warburton DER, Bauman A (2017). Physical activity: health impact, prevalence, correlates and interventions. Psychol Health.

[72] Schuch FB, Vancampfort D, Richards J, Rosenbaum S, Ward PB, Stubbs B (2016). Exercise as a treatment for depression: a meta-analysis adjusting for publication bias. J Psychiatr Res.

[73] Chater A (2017). HPPHN [Health Psychology in public health network]: look how far we have come in such a short time. Health Psychol Public Health.

[74] Chater A (2018). Developing and applying translational behavioural science to improve population health and reduce inequalities. Health Psychol Public Health.

[75] Howlett N, Jones A, Chater A (2019). Active Herts: translating behavioural science into public health. Behav Sci Public Health.

[76] Howlett N, Jones A, Bain L, Chater A (2017). How effective is community physical activity promotion in areas of deprivation for inactive adults with cardiovascular disease risk and/or mental health concerns? Study protocol for a pragmatic observational evaluation of the 'Active Herts' physical activity programme. BMJ Open.

[77] Michie S, van Stralen MM, West R (2011). The behaviour change wheel: a new method for characterising and designing behaviour change interventions. Implement Sci.

[78] Michie S, Richardson M, Johnston M, Abraham C, Francis J, Hardeman W, Eccles MP, Cane J, Wood CE (2013). The behavior change technique taxonomy (v1) of 93 hierarchically clustered techniques: building an international consensus for the reporting of behavior change interventions. Ann Behav Med.

[79] Howlett N, Schulz J, Trivedi D, Troop N, Chater A. A prospective study exploring the construct and predictive validity of the COM-B model for physical activity. J Health Psychol. 2019;24(10):1378–91. 10.1177/1359105317739098 29172808

[80] Howlett N, Trivedi D, Troop NA, Chater AM (2018). Are physical activity interventions for healthy inactive adults effective in promoting behavior change and maintenance, and which behavior change techniques are effective? A systematic review and meta-analysis. Transl Behav Med.

[81] NICE: Core components of a lifestyle weight management programme for overweight or obese adults. Weight management: lifestyle services for overweight or obese adults [https://pathways.nice.org.uk/pathways/lifestyle-weight-management-services-for-overweight-or-obese-adults#path=view%3A/pathways/lifestyle-weight-management-services-for-overweight-or-obese-adults/core-components-of-a-lifestyle-weight-management-programme-for-overweight-or-obese-adults.xml&content=view-node%3Anodes-weight-loss], accessed: [April 11, 2019].

[82] Chater A (2015). Motivational interviewing, health coaching and behaviour change. Enhancing communication skills for effective consultations. Training manual.

[83] McNeill A, Brose LS, Calder R, Bauld L, Robson D (2018). E-cigarettes and heated tobacco products: evidence review.

[84] Walker N, Howe C, Glover M, McRobbie H, Barnes J, Nosa V, Parag V, Bassett B, Bullen C (2014). Cytisine versus nicotine for smoking cessation. N Engl J Med.

[85] WHO (2019). WHO Report on the Global Tobacco Epidemic, 2019.

[86] McNeill A, Brose LS, Calder R, Bauld L, Robson DJ (2018). ArcbPHELPHE: Evidence review of e-cigarettes and heated tobacco products 2018.

[87] National Academies of Sciences, Engineering, and Medicine. Public health consequences of e-cigarettes. Washington: National Academies Press; 2018. 29894118

[88] Lancaster T, Stead LF. Self-help interventions for smoking cessation. Cochrane Database Syst Rev. 2005;3:CD001118. 10.1002/14651858.CD001118.pub2 16034855

[89] Brown J, Michie S, Geraghty AW, Yardley L, Gardner B, Shahab L, Stapleton JA, West R (2014). Internet-based intervention for smoking cessation (StopAdvisor) in people with low and high socioeconomic status: a randomised controlled trial. Lancet Respir Med.

[90] Ubhi HK, Michie S, Kotz D, Wong WC, West R (2015). A mobile app to aid smoking cessation: preliminary evaluation of SmokeFree28. J Med Internet Res.

[91] Kahlmeier S, Wijnhoven TM, Alpiger P, Schweizer C, Breda J, Martin BW (2015). National physical activity recommendations: systematic overview and analysis of the situation in European countries. BMC Public Health.

[92] Chater A. Behaviour change intervention design, delivery & evaluation: what you really need to know when developing services. Cranfield Local Health Authority, Bedfordshire 2016.

[93] Chater A. From design to evaluation… building an effective behaviour change intervention within public health: the ‘active Herts’ programme. Chisinau: Conference National Day of Health Promotion Specialist; 2018.

[94] Curtis K, Fulton E, K B (2018). Factors influencing application of behavioural science evidence by public health decision-makers and practitioners, and implications for practice. Prev Med Rep.

[95] Vega J (2013). Universal health coverage: the post-2015 development agenda. Lancet.

[96] WHO (2013). The world health report 2013: research for universal health coverage.

